# Insights into the Cytochrome P450 Monooxygenase Superfamily in *Kadsura heteroclita* (Xuetong)

**DOI:** 10.3390/molecules31122140

**Published:** 2026-06-17

**Authors:** Qian Xiao, Tianhao Fu, Mao Li, Ziyi Cai, Jiahui Yi, Jiaqi Liu, Mengqin Luo, Zhenni Xie, Chensi Tan, Jiang Zeng, Wei Wang, Luyun Ning

**Affiliations:** 1 School of Pharmacy, Hunan University of Chinese Medicine, Changsha 410208, China; 2 Traditional Chinese Medicine (TCM) and Ethnomedicine Innovation and Development International Laboratory, Hunan University of Chinese Medicine, Changsha 410208, China; 3 Institute of Materia Medica, Chinese Academy of Medical Sciences and Peking Union Medical College, Beijing 100050, China

**Keywords:** *Kadsura heteroclita*, cytochromes P450, Xuetongsu, bioinformatics analysis

## Abstract

*Kadsura heteroclita* (Roxb.) Craib, commonly known as “Xuetong”, is a traditional Tujia ethnomedicine with anti-rheumatoid arthritis (RA) activity, and schizanlactone E (Xuetongsu) is its major bioactive component whose biosynthetic pathway remains uncharacterized. As a cycloartane-type tetracyclic triterpenoid, Xuetongsu’s biosynthesis is likely to involve multiple oxidation steps. Cytochrome P450 (CYP450) is a versatile monooxygenase encoded by a large and diverse gene superfamily and plays a critical role in various oxidation reactions in plants’ secondary metabolism. In this study, 367 KhCYP450s were identified and systematically analyzed for their physicochemical properties, phylogenetic analysis, conserved motifs, gene structures, collinearity, and cis-acting elements. Weighted gene co-expression network analysis (WGCNA) revealed a turquoise module strongly associated with Xuetong root tissue, which had the highest Xuetongsu accumulation; 32 candidate *KhCYP450s* within this module were screened via correlation analysis between gene expression and xuetongsu content and partially validated by qRT-PCR. Five of these candidates showed significant homology with known triterpenoid biosynthetic genes via protein structure analyses. This study deepened our comprehension of the CYP450 superfamily in Xuetong and provided a valuable reference for further research on the biosynthesis of Xuetongsu.

## 1. Introduction

Cytochrome P450 (CYP450), a multifunctional enzyme family encoded by a complex gene superfamily, was the first gene family designated as a “superfamily” and constituted the largest enzyme family in plant metabolism [1,2]. As members of the heme-containing protein superfamily, CYP450s demonstrated versatile catalytic capabilities, principally facilitating oxidation and hydroxylation reactions that required molecular oxygen, with electron transfer mediated through NADPH-P450 reductase systems using NADPH as the electron donor [3,4]. CYP450s were ubiquitously present across cellular systems of plants, animals, bacteria, and fungi, and were predominantly associated with membrane structures of plastids, mitochondria, Golgi apparatus, and endoplasmic reticulum [5].

The nomenclature of cytochrome P450 was primarily based on amino acid sequence homology, with proteins sharing > 40% identity grouped within the same family and those with >55% identity categorized into identical subfamilies [6,7]. Phylogenetic analysis revealed that the CYP450 superfamily comprised 11 evolutionary clans, containing seven single-clan clusters (CYP51/74/97/710/711/727/746 clans) and 4 multifamily clusters (CYP71/72/85/86 clans) [6,8]. In plants, the CYP450 family was divided into A-type (represented by the plant-specific CYP71 clan) and non-A-type categories [9]. The non-A-type encompassed ten clans (CYP72/85/86/51/74/97/710/711/727/746 clans), exhibiting greater structural diversity and cross-kingdom conservation [10,11]. Advances in whole-genome sequencing enabled comprehensive characterization of CYP450s in model plants, including *Arabidopsis thaliana* and rice. To date, approximately 756,550 CYP450 sequences have been identified in the UniprotKB database (https://www.uniprot.org/), and over 30,000 CYP450s have been discovered and named in plants [12,13]. According to the Plant Cytochrome P450 Database (https://www.erda.dk/public/vgrid/PlantP450/table.html, accessed on 20 August 2025), more than 900 CYP450s were functionally characterized as capable of metabolizing one or more substrates [14].

*Kadsura heteroclita* (Roxb.) Craib was a traditional medicinal plant of the Tujia ethnic group in Xiangxi, China (vernacular name “Xuetong”), taxonomically belonging to the Schisandraceae family and the genus *Kadsura* [15,16,17]. It was well documented to dispel wind-dampness, nourish blood, promote blood circulation, regulate Qi, and alleviate pain [18,19]. Its roots and stems were empirically used for the treatment of rheumatoid arthritis (RA), Qi-related pain, epigastric pain, lumbar muscle strain, dysmenorrhea, ostealgia, common cold, and postpartum paralysis [15,16,17]. Modern pharmacological studies confirmed that Xuetong-derived extracts exhibited anti-RA, anti-inflammatory, analgesic, anti-HIV, antitumor, and hepatoprotective properties [15,20,21,22,23]. A diverse array of secondary metabolites, including lignans, triterpenoids, and sesquiterpenoids, were isolated and characterized from Xuetong. Many of these compounds exhibited a high degree of oxidation, featuring hydroxylation or carboxylation modifications, such as Xuetongsu, xuetonin C, xuetonlignans D, and 6α,9α,15-trihydroxycadinan-4-en-3-one [21,24,25,26,27,28]. Notably, Xuetongsu (Schisanlactone E), the primary component of Xuetong stem, was identified as a cycloartane-type tetracyclic triterpene exhibiting anti-inflammatory, analgesic, and anti-RA pharmacological activities and served as the primary active ingredient responsible for Xuetong’s therapeutic efficacy in RA. Furthermore, its significant hepatoprotective effects and exceptionally low toxicity established this compound as a promising candidate for RA therapy. Recent experimental studies successfully conjugated Xuetongsu with Prussian blue nanoparticles (PB NPs), enabling targeted RA treatment while maintaining a favorable safety profile and enhanced therapeutic efficacy [29]. However, its content in Xuetong was low, and the structure was complex which made its chemical synthesis difficult [30]. Moreover, the biosynthetic pathway of Xuetongsu has not yet been reported, which severely restricts its further application. With the rapid development of molecular biology techniques and the increasing interest in the study of ethnic medicinal plants, research on the biosynthesis of major active components in Xuetong and other ethnic medicinal plants will receive extensive attention.

In plants, CYP450s were mainly involved in the biosynthesis of various primary and secondary metabolites, including terpenoids, alkaloids, lipids, glycosides and plant hormones, or played roles in detoxification and other stress resistance processes [3,31]. In vitro enzymatic experiments demonstrated that *CYP5150L8* mediated the three-step C-26 oxidation (hydroxylation, aldehyde formation, and carboxylation) of lanosterol to generate ganoderic acid and 3-hydroxy-lanost-8,24-dien-26-oic acid (HLDOA). Furthermore, heterologous expression of *CYP5150L8* in *Saccharomyces cerevisiae* enabled the biosynthesis of HLDOA [32]. Subsequent studies identified *CYP5139G1* as catalyzing the C-28 oxidation of HLDOA, resulting in a novel ganoderic acid, 3,28-dihydroxy-lanosta-8,24-dien-26-oic acid (DHLDOA) [33]. An investigation first revealed the key gene cluster (*CYP87G1*, *CYP88C7*, *CYP749B2*) responsible for withanolide biosynthesis in Solanaceae plants. *CYP87G1* hydroxylated 24-methyl-cholesta-5,24-dien-3-one at C-22 to yield (22R)-ergosta-5,24-dien-3β,22-diol; *CYP88C7* introduced a hydroxyl group at C-1 of this compound to form (22R)-ergosta-5,24-dien-1α,3β,22-triol; and *CYP749B2* catalyzed δ-lactone ring formation between C-22 and C-26 on the side chain of (22R)-ergosta-5,24-dien-1α,3β,22-triol, thereby producing withanoside V [34]. Additionally, *CsCYP716AC1* was shown to mediate Baeyer–Villiger oxidation of 21-(S)-acetoxyl-apo-melianone, generating the A-ring lactone [35].

**Figure 1:**
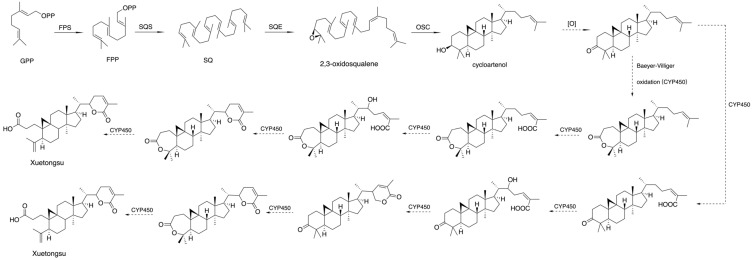
The hypothesized biosynthetic pathway of Xuetongsu.

Based on proposed biosynthetic pathways in the literature, Xuetongsu—a tetracyclic triterpenoid—was suggested to be derived from cycloartenol as its precursor [36] and its hypothesized biosynthetic pathway was presented in Figure 1. CYP450s were implicated in catalyzing the A-ring lactonization via Baeyer–Villiger oxidation and concurrently mediating side-chain oxidation of cycloartenol, including hydroxylation at the C-22 position and sequential oxidative modifications at C-26 that generate hydroxyl, aldehyde, and carboxyl intermediates. Subsequently, the functional groups at C-22 and C-26 were proposed to undergo either enzyme-catalyzed or spontaneous intramolecular cyclization to form the characteristic lactone ring side chain of Xuetongsu, thereby contributing to its biosynthesis.

Current research on Xuetong has predominantly focused on its chemical composition, pharmacological activities, and the mechanistic basis of therapeutic efficacy. However, comprehensive investigations addressing genome-wide analysis of CYP450 in Xuetong remained unreported. This study aimed to perform systematic bioinformatics analysis of the *CYP450* gene family (*KhCYP450s*) in Xuetong and screen key *KhCYP450* genes potentially involved in Xuetongsu biosynthesis. This study provided valuable insights into the CYP450 superfamily and established a foundation for future research on Xuetongsu biosynthesis.

## 2. Results

### 2.1. Identification of KhCYP450s

**Figure 2:**
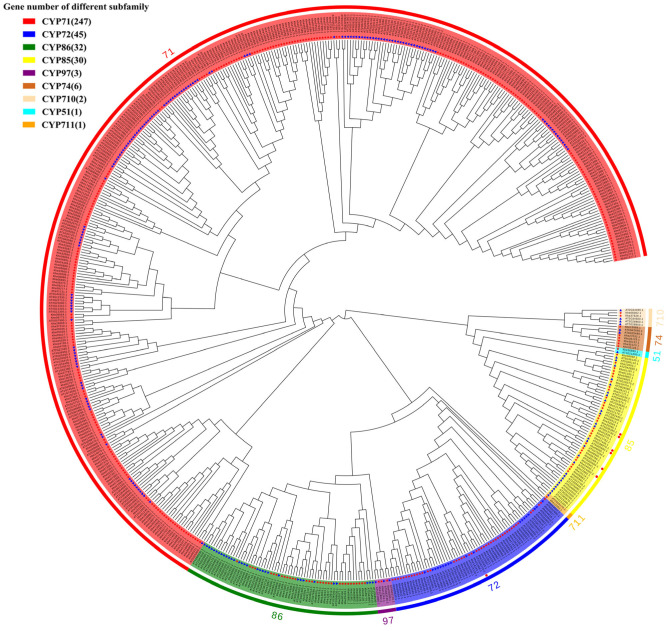
Phylogenetic analysis of the KhCYP450s and AtCYP450s. KhCYP450s were indicated by red stars, and AtCYP450s were labeled by blue triangles. The seven functionally characterized AtCYP450s involved in triterpenoid and steroid biosynthesis were highlighted with red dots. The outermost numbers in different colors showed different clans.

The CYP450 superfamily members were systematically identified in the Xuetong genome using HMMER with the Pfam CYP450 domain (PF00067) as a query, yielding 449 preliminary candidate transcripts (Table S1). Following the filtration of alternative splicing-derived redundancy and validation via CYP450 conserved domain architecture analysis, 367 non-redundant *KhCYP450* genes were retained (Figure 2; Table S2; Figure S1). Extensive physicochemical characterization revealed substantial molecular diversity: protein lengths ranged from 102 (Khe10168.1) to 1332 amino acids (Khe33589.1), with molecular weights spanning from 11.52 to 146.59 kDa. The pI values exhibited a range from 4.5 (Khe07364.1) to 10.42 (Khe05471.1). Among these, 265 proteins exhibited pI values greater than 7 and were categorized as basic proteins. Khe15856.1, Khe15865.1, and Khe15867.1 displayed pI values equal to 7 and were classified as neutral. The remaining 99 proteins had pI values below 7 and were considered acidic proteins. Instability indices ranged from 19.25 to 59.74, classifying 155 proteins as unstable (<40), while aliphatic indices ranged from 66.73 to 113.01 and the grand average of hydropathy ranged from −0.637 to 0.233. Only 45 proteins had a grand average of hydrophilicity greater than 0, which were classified as hydrophobic proteins.

Subcellular localization analysis indicated that the majority of KhCYP450s were localized to the endoplasmic reticulum (281 out of 367), comprising 138 transmembrane proteins and 143 peripheral membrane proteins. Among these 281 proteins, 275 were predicted to contain signal peptides, and 205 were predicted to possess one to three transmembrane helices at their N-terminus. This structural feature may facilitate anchoring to the endoplasmic reticulum membrane and promote precise docking between functional domains of KhCYP450s and their substrates. The second most prevalent localization was the cytoplasm (36 out of 367). The smallest number of KhCYP450s was identified in the peroxisome, with only one protein localizing to this compartment. Detailed gene annotations and protein characteristics are provided in Table S2.

### 2.2. Phylogenetic Analysis of KhCYP450s

To further elucidate the phylogenetic and molecular evolutionary relationships among KhCYP450s, 367 KhCYP450 protein sequences were aligned with 235 AtCYP450s of *A. thaliana* (Table S3), and a phylogenetic tree was constructed using the maximum likelihood method. Based on the established classification system of AtCYP450 proteins [37], all CYP450 proteins were categorized into nine distinct clans. The KhCYP450 proteins were further classified into two major clades according to their phylogenetic positions: A-type (CYP71 clan) and non-A-type clusters encompassing eight clans (CYP51/72/85/86/97/710/711/74 clans). The largest cluster was the CYP71 clan, comprising 247 KhCYP450 proteins; the smallest clusters were the CYP51 and CYP711 clans, each containing one member (Figure 2; Table S2).

To correlate these classifications with known functional data, seven experimentally validated *A. thaliana* triterpenoid and steroid biosynthesis-related CYP450s were mapped onto the phylogenetic tree (Figure 2; Table S4) [38,39]. These genes mainly clustered in the CYP85 clan, with only one member in the CYP72 clan. Additionally, CYP71, CYP72, and CYP85 were collectively recognized as the major functional clans responsible for triterpenoid- and steroid- modification [38]. Among these reference genes, AT3G50660.1 and AT5G14400.1 mainly participated in steroid C-22 hydroxylation and were phylogenetically close to KhCYP35269.1, KhCYP22862.1 and KhCYP10114.1. AT2G26710.1 mediated C-26 hydroxylation of castasterone and brassinolide, with KhCYP01968.1 and KhCYP14242.1 located on adjacent phylogenetic branches. These closely related KhCYP450s were likely to share similar catalytic functions in triterpenoid and steroid metabolism.

### 2.3. Conserved Motifs and Gene Structures of KhCYP450s

**Figure 3:**
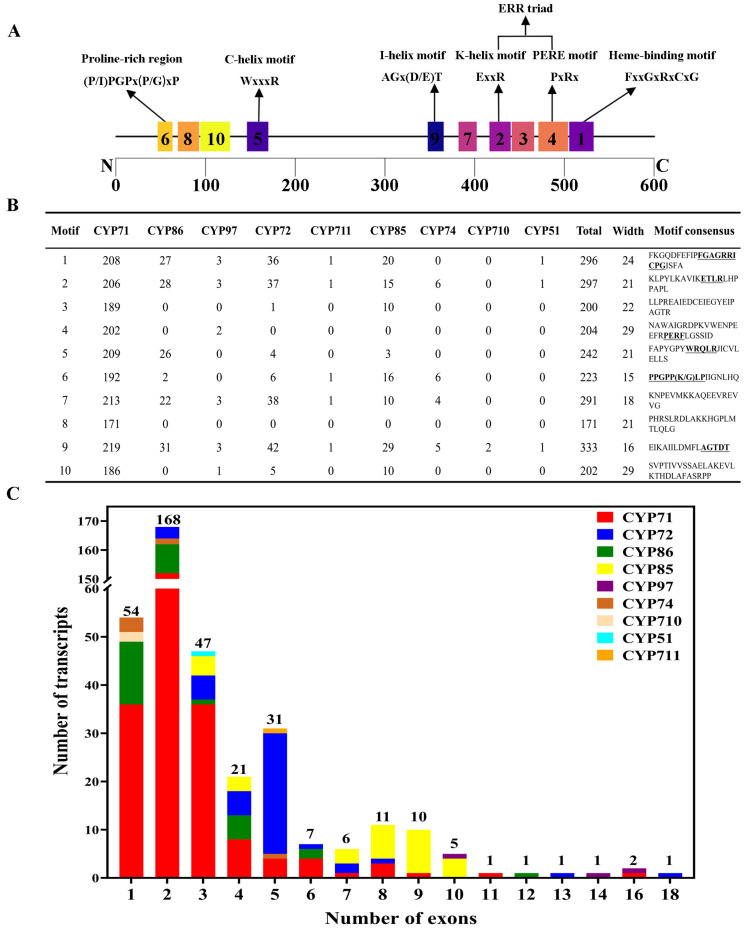
Distribution of conserved motifs and exons in 9 clans of KhCYP450s. (**A**) Schematic representation of 10 conserved motifs identified in KhCYP450s, highlighting those associated with functionally characterized domains. N and C denote the N-terminal and C-terminal regions, respectively. (**B**) Summary of the 10 conserved motifs across the 9 KhCYP450 clans. (**C**) Exon distribution patterns of transcripts within the 9 KhCYP450 clans.

A total of 10 conserved motifs (motifs 1–10) were identified across KhCYP450 proteins, with lengths ranging from 16 to 29 amino acids. All these 10 motifs were detected in the A-type KhCYP450s, whereas only a limited number of motifs were observed in non-A-type KhCYP450s, particularly in the CYP711, CYP710, and CYP51 clans (Figure 3A,B, Figures S2 and S3). Among these motifs, motifs 1, 2, 6, 7, and 9 displayed high conservation, with motif 9 present across all CYP450 family clusters. In contrast, the remaining five motifs were frequently absent in non-A-type members, while motifs 4 and 8 were predominantly found in A-type CYP450s (Figure 3B). Notably, six motifs (motif 1, 2, 4, 5, 6, and 9) were located within functionally characterized domains (Figure 3B).

Analysis of the gene structure of *KhCYP450s* (Figure 3C and Figure S4; Table S2) revealed that the distribution of CDS, UTR, introns, and exons exhibited clan-specific patterns. Notably, the structural composition of CDS-UTR regions differed markedly between A-type and non-A-type *KhCYP450s*, whereas members within the same clan were relatively similar (Figure S4). A-type *KhCYP450s* generally contained fewer introns, while non-A-type *KhCYP450s*—particularly those in the CYP97, CYP85, and CYP72 clans—typically possessed a higher number of introns (Table S2). Furthermore, the exon numbers of *KhCYP450s* displayed considerable variation (Figure 3C; Table S2), ranging from 1 to 18. Specifically, A-type *CYP450s* possessed between 1 and 16 exons, predominantly falling within the range of 1 to 8 exons. In contrast, non-A-type *CYP450s* exhibited greater variability in exon number, spanning the full range from 1 to 18. For example, the CYP85 clan contained 3 to 10 exons; the CYP710 clan had only 1 exon; the CYP72 clan displayed a broad range of 2 to 18 exons; and the CYP74 clan generally contained 1–5 exons. Notably, *Khe15889.1* was the only member in the CYP74 clan with five exons, while all other members possessed either one or two exons. Furthermore, the exon numbers within the CYP74 clan mirrored the branching patterns of its genes in the phylogenetic tree.

In summary, A-type and non-A-type CYP450s exhibited distinct differences in conserved motif architecture and gene structural composition. However, CYP450 proteins within the same clan displayed broadly conserved patterns in both motif distribution and gene structure.

### 2.4. Chromosomal Localization, Duplication Events and Synteny Analysis of KhCYP450s

The chromosomal localization of *KhCYP450s* was presented in Figure S5. A total of 348 *KhCYP450s* were found to be unevenly distributed across 14 chromosomes, while the remaining 19 genes remained undetermined due to gaps in the genome assembly. Chromosome 11 harbored the largest number (42 members), followed by chromosome 2 with 37 members. Chromosomes 7 and 14 contained the fewest *KhCYP450* sequences, possessing 10 and 12 genes, respectively.

**Figure 4:**
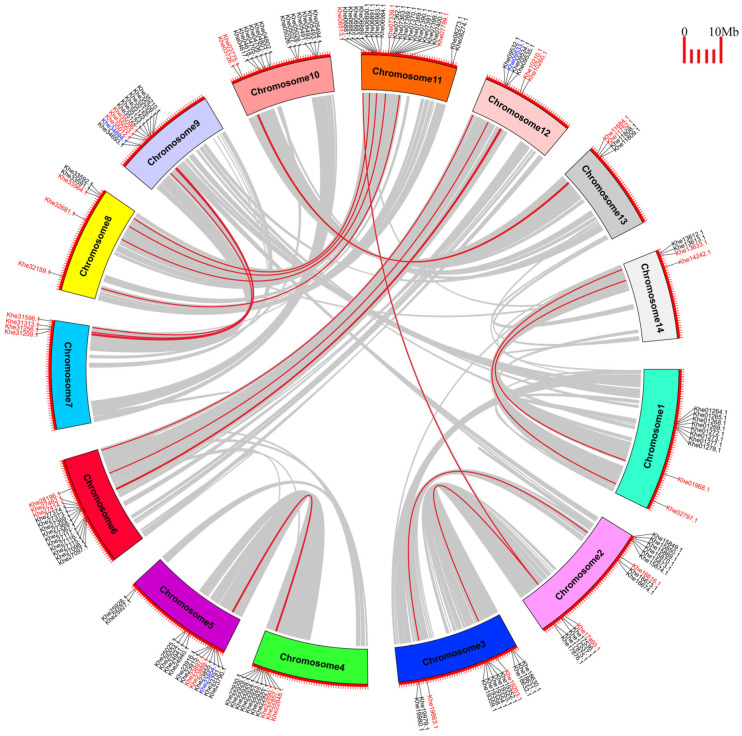
Synteny analysis of *KhCYP450s*. The gray lines in the innermost circle depicted all homologous segments identified in Xuetong, whereas the highlighted red lines represented 19 WGD or segmentally duplicated gene pairs within the *KhCYP450s* family. The fourth circle represented the 14 chromosomes of Xuetong. On the outermost layer, duplicated *KhCYP450* genes were annotated: Tandem duplications were labeled in black, WGD or Segmental duplications in red, and genes exhibiting both duplication types were marked in blue. The scale above each chromosome label indicates the chromosomal positions of *KhCYP450* genes.

Synteny analysis classified the evolutionary origins of *KhCYP450s* into four duplication types: Dispersed, Tandem, Proximal, and WGD or Segmental. Among these types, a total of 114 *KhCYP450s* (31.06%) were attributed to tandem duplication events (Figure 4; Table S2). Of these, five arose from heterologous tandem duplications between *KhCYP450s* and non-*KhCYP450* genes, while the remaining 109 resulted from intrafamily tandem duplications within the *KhCYP450* family, forming 62 tandem duplicated gene pairs. Based on their copy number and genomic position, these tandem duplicates were categorized into three major groups (containing 2, 3 or 4 genes) and 47 minor groups: comprising 35 minor groups with two genes, nine with three genes, and three with four genes (Tables S5 and S6). Among the 14 chromosomes of Xuetong, Chromosome 11 harbored the highest number of tandem duplicated genes (*n* = 19), whereas no tandem duplications were detected on Chromosome 7.

Additionally, 42 *KhCYP450* genes (11.44%) were derived from WGD or segmental duplication events (Figure 4; Table S2), four of which involved duplications between *KhCYP450s* and non-*KhCYP450* genes. Notably, *Khe06881.1* originated from both tandem and WGD or Segmental duplication mechanisms. The remaining 38 genes were classified as intrafamily WGD or segmental duplications, forming 19 WGD or segmental duplicated gene pairs (Table S6). Three genes (*Khe09533.1*, *Khe23854.1*, and *Khe34694.1*) possessed dual evolutionary origins, with evidence supporting both tandem and WGD or segmental duplication events. Comparative analysis of Ks values (larger Ks values denote older duplication episodes) revealed that the WGD or segmental duplication events in these three genes predated the tandem duplication events. Moreover, among all duplicated gene pairs in the *KhCYP450* family, only the pair *Khe16673.1* and *Khe16674.1* exhibited a Ka/Ks ratio greater than 1. In contrast, almost all other duplicated gene pairs displayed Ka/Ks ratios below 1.

Dispersed and Proximal duplications accounted for 25.07% (*n* = 92) and 23.71% (*n* = 87) of the *KhCYP450s*, respectively. Another 36 *KhCYP450* genes could not be categorized into any of the four duplication modes. Duplication classification and Ka/Ks statistics uncovered diverse evolutionary expansion patterns underlying the *KhCYP450* gene family.

### 2.5. Analysis of Cis-Acting Elements in the Promoter Region of KhCYP450s

A total of 74 distinct cis-acting elements were identified and classified into eight functional categories: Optical bonding elements, Protein binding sites, Hormone responsive elements, Metabolic regulatory elements, Anti-reverse adjustment elements, Tissue-specific regulatory elements, Cell cycle rhythm regulatory elements, and Promoter action elements (Figures S6 and S7; Tables S7 and S8). Minimal variation in cis-acting element composition was observed across different CYP450 clans in Xuetong. Notably, Optical bonding elements were the most abundant, with each *KhCYP450* gene containing 1–12 distinct types. The five most prevalent motifs included G-Box (76.5%), Box 4 (61.8%), GT1-motif (57.7%), TCT-motif (57.7%), and GATA-motif (47.1%). Additionally, 358 *KhCYP450s* contained 1–5 types of anti-reverse adjustment elements associated with drought, low temperature, or other environmental stresses. Among these, the hypoxia-inducible element ARE was the most widely distributed, which occurred in the promoters of 320 (87.1%) *KhCYP450s* promoters. Hormone-responsive elements were predominantly associated with five hormones, including methyl jasmonate (MeJA), abscisic acid, gibberellin, and others. Among these, a total of 247 *KhCYP450* genes contained between 2 and 26 MeJA-responsive elements. Furthermore, multiple development-associated regulatory elements were predicted, including the cell cycle-regulated MSA-like element (5.7%), the meristem-specific expression element CAT-box (67.5%), and the O2-site motif involved in zein metabolism regulation (38.4%). Overall, although no subfamily-specific distribution patterns of cis-acting elements were evident, the diversity of these cis-acting elements indicated that *KhCYP450* expression was influenced not only by light, hormonal signals, and environmental stressors, but also potentially modulated by various developmental processes.

### 2.6. Gene Function Annotation of KhCYP450s

**Figure 5:**
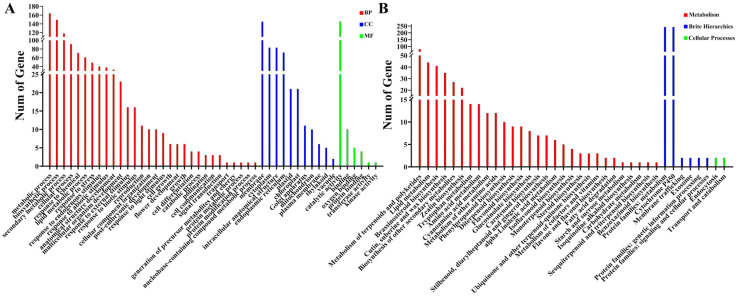
GO and KEGG annotation of *KhCYP450s*. (**A**) The GO terms annotation of *KhCYP450s*; (**B**) The KEGG pathway annotations of *KhCYP450s*.

Gene Ontology (GO) analysis identified a total of 47 GO terms associated with *KhCYP450s* (Figure 5A; Table S9), which were classified into three major functional categories: Biological Process (BP), Cellular Component (CC), and Molecular Function (MF). Within the BP category, 30 terms were enriched, predominantly associated with metabolic processes (GO:0008152). The CC category encompassed 11 terms, with membrane structures (GO:0016020) being the most highly represented. In the MF category, six terms were identified, primarily enriched in catalytic activity (GO:0003824). Kyoto Encyclopedia of Genes and Genomes (KEGG) pathway annotation revealed 37 significantly enriched pathways among *KhCYP450s* (Figure 5B; Table S10), grouped into three main sections: Metabolism, Brite Hierarchies, and Cellular Processes. Among the 29 metabolism-related pathways, most pathways were involved in the biosynthesis and metabolism of secondary metabolites such as terpenoids and polyketides, as well as primary metabolites, including amino acids and tryptophan. Notably, *Khe27368.1* was simultaneously mapped to both the “Metabolism of terpenoids and polyketides” and “Sesquiterpenoid and triterpenoid biosynthesis” pathways, suggesting its potential role in terpenoid modification. The six Brite Hierarchies entries primarily encompassed CYP450 and metabolism-related protein families. Additionally, two pathways related to Cellular Processes were identified—endocytosis as well as transport and catabolism—highlighting potential roles in cellular trafficking and degradation. GO and KEGG enrichment results collectively indicated that *KhCYP450* members were mainly annotated to metabolite synthesis pathways, especially triterpenoid metabolic routes.

### 2.7. WGCNA of KhCYP450s

Gene expression typically exhibits variations across different tissues owing to functional specificity. To investigate the expression patterns of the *KhCYP450* gene family, transcriptomic analysis and WGCNA were performed on six distinct tissues of Xuetong, including roots, leaves, fruits, bark, xylem, and flowers.

**Figure 6:**
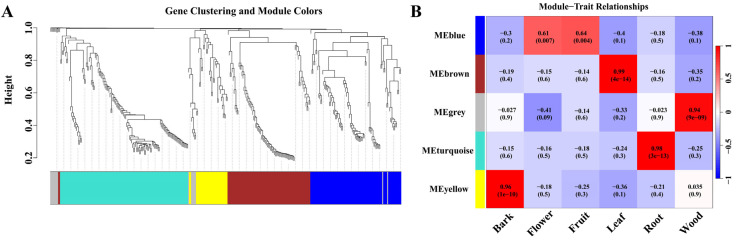
WGCNA of *KhCYP450s* across different tissues in Xuetong. (**A**) Co-expression modules of *KhCYP450s*. Each terminal node represented an individual gene; The major tree branches constitute 5 modules labeled with different colors. (**B**) Module-trait correlation matrix based on Pearson correlation coefficients. The colored bars on the left indicated distinct co-expression modules, and the numerical values within the figure denoted the correlation coefficients between modules and traits, along with their associated *p* values. The color gradient from −1 (blue) to 1 (red) on the right reflected the strength and direction of the correlations.

As shown in Figure 6A, five co-expression modules were ultimately detected from the clustering dendrogram. The turquoise module contained the highest number of genes (*n* = 130), whereas the gray module contained the fewest (*n* = 16) (Table S11). Pearson correlation analysis was used to assess the associations between these modules and tissue types, with results visualized as a heatmap of the module-trait correlation matrix (Figure 6B). Among the five modules, four showed strong correlations with specific tissues (*p* < 0.001): the turquoise, brown, yellow, and gray modules exhibited significant associations with root (*R* = 0.98), leaf (*R* = 0.99), bark (*R* = 0.96), and wood (*R* = 0.94), respectively. Xuetongsu, a major bioactive component of Xuetong, was predominantly accumulated in roots when compared to other tissues (Figure S8). This suggested that genes within the root-associated turquoise module might be involved in the biosynthetic pathway of Xuetongsu. Therefore, the turquoise module was selected as the candidate module for further investigation.

### 2.8. Verification by qRT-PCR

To further explore the association between the expression of *KhCYP450* genes and Xuetongsu accumulation, we performed Spearman correlation analysis between *KhCYP450s* transcript abundance and Xuetongsu content. The results showed that most *KhCYP450s* were positively correlated with Xuetongsu accumulation. Specifically, 35 *KhCYP450s* exhibited a strong correlation (*R* > 0.75, *p* < 0.01) (Tables S12 and S13), among which 32 predominantly clustered within the turquoise module. The 32 turquoise module genes were assigned CYP71 (*n* = 25), CYP72 (*n* = 4), CYP74 (*n* = 1), and CYP85 (*n* = 2) clans.

**Figure 7:**
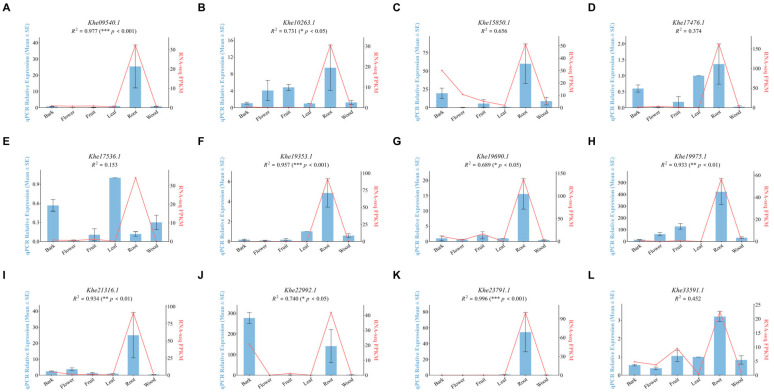
qRT-PCR validation of candidate *KhCYP450s* associated with Xuetongsu metabolism. Blue bars represented relative expression levels quantified by qRT-PCR (mean ± SE, *n* = 3), and red lines indicated corresponding FPKM values from RNA-seq data. Pearson’s determination coefficient (*R*^2^) and *p*-value were calculated to assess expression consistency. Asterisks represent statistically significant differences: * indicates *p* < 0.05, ** indicates *p* < 0.01, and *** indicates *p* < 0.001.

To verify the reliability of transcriptomic expression data, we randomly selected 12 *KhCYP450* genes from these 32 genes for qRT-PCR expression detection across different tissues. Pearson correlation analysis was applied to assess the consistency between qRT-PCR relative expression levels and RNA-seq FPKM values. The verification results presented obvious hierarchical differences in expression consistency among these detected genes. Five genes, namely *Khe09540.1*, *Khe19353.1*, *Khe19975.1*, *Khe21316.1* and *Khe23791.1*, showed an extremely high consistency (*R*^2^ > 0.9, *p* < 0.01) (Figure 7; Table S14). Another three genes (*Khe10263.1*, *Khe19690.1*, *Khe22992.1*) showed moderate but statistically significant correlation, with *R*^2^ values ranging from 0.689 to 0.740 (*p* < 0.05). The remaining four genes showed no significant correlation between qRT-PCR and transcriptomic data, with inconsistent expression trends.

These divergent verification results effectively distinguished genes with stable expression traits from those with fluctuating expression patterns, avoiding the limitation that all candidate genes shared identical expression trends. In summary, the eight genes with stable and reproducible tissue expression patterns were regarded as promising candidates potentially involved in Xuetongsu biosynthesis.

### 2.9. Analysis of Protein Structure

To further investigate the potential involvement of candidate *KhCYP450* genes in Xuetongsu biosynthesis, we performed BLAST analyses of the eight protein sequences against the UniProt database. As shown in Table S15, five of these proteins exhibited high similarity to functionally characterized triterpenoid-biosynthetic CYP450s: Khe19353.1 and Khe23791.1 matched CYP71BQ subfamily members (CYP71BQ17 and CYP71BQ5, respectively) with identity scores of 66.3% and 63.9%; Khe09540.1 and Khe21316.1 aligned to CYP72A154 and CYP93E1, showing 65.6% and 61% identity. Notably, Khe22992.1 (encoded by one of the three moderately correlated genes) shared 62.7% identity with CYP71CD4; all these matches had E-values < 1 × 10^−100^, confirming reliable sequence homology. In contrast, Khe19975.1, Khe10263.1, and Khe19690.1 showed low similarity to triterpenoid-biosynthetic CYP450s, indicating insufficient sequence conservation for reliable structural and functional prediction. Thus, they were not included in subsequent structural analysis.

Comparative structural analysis was conducted by retrieving AlphaFold-predicted PDB templates of the five triterpenoid-related reference enzymes and constructing 3D models of their corresponding KhCYP450s (Khe09540.1, Khe19353.1, Khe21316.1, Khe23791.1, Khe22992.1) via AlphaFold3. All models generated high-confidence 3D structures, with pTM scores ranging from 0.87 to 0.93. Structural validation through Swiss-Model revealed robust model quality parameters: QMEANDisCo Global scores > 0.6 and Ramachandran favored residues occupying 96.96–99.22% of structural positions (Table S16), confirming the reliability of these models for subsequent functional characterization.

**Figure 8:**
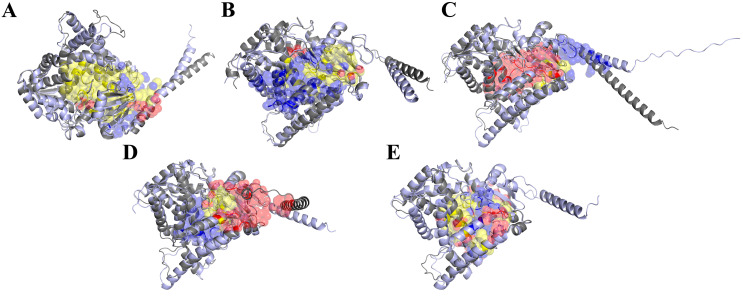
Structural comparisons of KhCYP450s with their respective reference CYP450s. (**A**) Khe09540.1 and CYP72A154; (**B**) Khe21316.1 and CYP93E1; (**C**) Khe19353.1 and CYP71BQ17; (**D**) Khe23791.1 and CYP71BQ5; (**E**) Khe22992.1 and CYP71CD4. All structures are displayed in cartoon representation, with KhCYP450s in gray and reference CYP450s in light blue. Predicted binding pockets were depicted as spheres: red for KhCYP450s, blue for reference proteins, and yellow for overlapping regions within a 3 Å distance threshold. Detailed data were presented in Table S15.

Active site prediction by CASTpFold, coupled with PyMOL structural visualization, was used to assess the conservation of catalytic regions between each of the five KhCYP450s and their corresponding triterpenoid-biosynthetic reference enzymes. Root mean square atomic deviation (RMSD) values of their active sites ranged from 0.918 to 1.402 Å (Table S15), indicating overall spatial similarity. Comparative analysis of active site architectures revealed notable differences among the five KhCYP450-reference enzyme pairs: Khe09540.1 (CYP72A154 homolog) and Khe21316.1 (CYP93E1 homolog) showed strong structural convergence, exhibiting centroid distances of <10 Å and spatial overlap ratios of 81.82% and 74.41%, respectively; Khe22992.1 (CYP71CD4 homolog) displayed moderate structural conservation, with a very close pocket center distance (2.581 Å) but a moderate overall overlap ratio (57.12%); in contrast, Khe23791.1 (CYP71BQ5 homolog) and Khe19353.1 (CYP71BQ17 homolog) showed substantial active site displacements, with pocket center distances of 19.764 Å and 22.074 Å, and spatial overlap ratios < 30% (Figure 8; Table S15). Quantitative pocket parameters demonstrated distinct degrees of active-site structural conservation among the five candidate CYP450 proteins.

## 3. Discussion

### 3.1. Comprehensive Identification of the KhCYP450 Genes

The number of *CYP450* genes exhibited significant variation among different plant species. In *citrus*, 301 *CYP450* genes were identified and classified into 10 clans [11]. Rice contained 326 genes encoding 355 CYP450 proteins, which were grouped into 10 clans [40]. In *Osmanthus fragrans*, a total of 276 *CYP450* genes were identified and assigned to 7 clans [41]. Similarly, *Taxus chinensis* was found to possess 293 *CYP450* genes organized into 8 clans [42]. In this study, 367 *CYP450* genes were identified in Xuetong, which were unevenly distributed across 14 chromosomes and classified into 9 distinct clans through phylogenetic analysis. Compared with most reported medicinal woody plants, the expanded gene number implied lineage-specific family expansion, which presumably supported the diversified biosynthesis of triterpenoids in Xuetong.

Six functionally characterized motifs (1, 2, 4, 5, 6, and 9) were identified, with all except motif 6 demonstrating direct or indirect involvement in heme-enzyme binding interactions essential for catalytic activity. Motif 1 contained the conserved heme-binding signature (FxxGxRxCxG), a hallmark CYP450 feature observed in 296 KhCYP450 proteins. Cysteine (C) in this motif acted as the fifth axial ligand to heme iron. In its thiolate form, it promoted formation of a ferrous carbon monoxide complex, producing the characteristic 450 nm absorption peak [11,43]. Motif 2 included the helix K motif (ExxR), where glutamic acid (E) and arginine (R) interacted with the arginine residue in the PERF motif (PxRx) located within motif 4, forming an E-R-R triad [44]. This interaction generated a stable salt bridge effect that stabilized the heme pocket structure and maintained the integrity of the heme binding site. Motif 5 harbored the helix C motif (WxxxR), where tryptophan (W) and arginine (R) bound heme propionates for proper heme coordination [11,45]. Motif 6 occurred in 223 KhCYP450s, with the sixth amino acid residue displaying comparable frequencies of lysine (K) and glycine (G), supporting the characterization of the (P/I)PGPx(P/G)xP sequence. This region was classified as a membrane hinge due to its high proline (P) and glycine (G) content, hydrophobicity, and structural flexibility, and has been demonstrated to play a critical role in CYP450 membrane localization [46]. All KhCYP450s containing this motif were localized to the endoplasmic reticulum or plastids, consistent with and further validating subcellular localization predictions. Motif 9 encompassed the conserved helix I motif (AGxDT), essential for heme-dependent oxygen activation via proton channel formation [45]. These findings suggested that KhCYP450s containing these motifs possessed higher catalytic potential, with CYP71 and CYP72 subfamilies exhibiting particularly high retention rates of these functional motifs. Notably, subfamily-specific distribution patterns were observed where N-terminal motifs 8/10 and C-terminal motifs 3/4 demonstrated CYP71 clan enrichment. Their frequent absence in non-A-type CYP450s aligned with the evolutionary hypothesis that these older non-A-type CYP450s developed diversified structural configurations through prolonged genetic reorganization and duplication events [10]. This structural polymorphism likely underlay the substrate specificity mechanisms and functional versatility observed in CYP450-mediated biological processes.

In plants, CYP450s catalyzed diverse biochemical processes and played a significant role in multiple biological processes, including stress responses and growth development [47,48]. For instance, CYP71D8L modulated rice growth and stress responses by regulating the homeostasis of phytohormonal gibberellin and cytokinin [49]. In *citrus* species, the UV-B-induced CYP gene *CitF3′H* within light signaling pathways was demonstrated to regulate 3′-hydroxylated flavonoid biosynthesis [11]. In *Cajanus cajan*, CcCYP75B165, strongly induced by MeJA, was found to potentially play important roles in the biosynthesis of flavonoids [50]. Furthermore, this study revealed that the MeJA-responsive cis-acting element was present in the promoter regions of the majority of *KhCYP450* genes. Previous studies have demonstrated that MeJA treatment upregulates the expression of genes involved in the triterpenoid biosynthesis pathway in Xuetong to varying degrees, indicating a clear inductive effect [51]. These findings suggested that *KhCYP450s* containing this cis-acting element are likely involved in the biosynthesis of triterpenoids and potentially of Xuetongsu, while definitive catalytic evidence required further functional verification.

### 3.2. KhCYP450s Involved in the Biosynthesis of Xuetongsu

Structural analysis suggested that Xuetongsu biosynthesis likely involved multiple oxidative modifications (Figure 1). In this study, 32 genes showed particularly strong correlations with Xuetongsu content and were assigned to the CYP71, CYP72, CYP74, and CYP85 clans. This classification was consistent with previous phylogenetic evidence that CYP71, CYP72 and CYP85 serve as core clans for triterpenoid and steroid biosynthesis in *A. thaliana*. Nevertheless, xuetongsu-related candidates were mainly enriched within the CYP71 clan, indicative of distinct lineage-specific divergence relative to *A. thaliana* steroid-synthesizing CYP450s. Additionally, eight of them displayed stable and consistent expression trends between RNA-seq and qRT-PCR results, whereas another four showed inconsistent expression patterns. Such discrepancies were likely mainly ascribed to divergent normalization strategies: RNA-seq-derived FPKM values adopted global transcriptome normalization, whereas qRT-PCR data were normalized via internal reference genes. This methodological difference tended to cause systematic deviations, particularly for low-expression CYP450 genes. In addition, distinct CYP450 members showed varied sensitivity to subtle individual physiological variations and minor differences in tissue development, which could induce unstable expression fluctuations.

CYP450s were widely documented as essential enzymes participating in plant terpenoid biosynthesis. For example, CYP71A16 catalyzed the C-23 hydroxylation during marneral biosynthesis in *A. thaliana* [52]. Concurrently, CYP72A63 was primarily associated with the C-30 oxidation of β-amyrin biosynthesis in *Medicago truncatula* [53]. Our protein structure analysis identified five triterpenoid-associated genes exhibiting high homology. The CYP72A154, the homolog Khe09540.1, was identified to catalyze three successive C-30 oxidations of 11-oxo-β-amyrin (hydroxylation, aldehyde formation, carboxylic acid formation) to produce glycyrrhetinic acid in *Glycyrrhiza* species [53]. The CYP93E1 (the homolog of Khe21316.1) predominantly facilitated C-24 hydroxylation of β-amyrin and sophoradiol in *Glycine max* [54]. The CYP71CD4 (homolog of Khe22992.1) catalyzed the conversion of tirucalla-7,24-dien-3β-ol to dihydroniloticin via C-23 hydroxylation and C-24-25 epoxidation [55]. Moreover, CYP71BQ17 (the homolog of Khe19353.1) in *Ailanthus altissima* [55] and CYP71BQ5 (the homolog of Khe23791.1) in *Melia azedarach* [56] exhibited catalytic specificity for C-21 hydroxylation of tirucalla-7,24-dien-3β-ol. Combined with structural analysis, the Khe09540.1, Khe21316.1 and Khe22992.1 showed their catalytic activities were likely conserved due to their short centroid distances and high overlap ratios. In contrast, the active site displacements and low spatial overlap ratios in the Khe23791.1 and Khe19353.1 implied potential distinct substrate specificities and catalytic mechanisms, despite the conservation of their overall structure. These collective findings indicated that the CYP71 and CYP72 clans were critical in Xuetong triterpenoid metabolism. The evolutionary conservation observed in *KhCYP450s* implied analogous functional potential in Xuetongsu biosynthesis.

However, none of these triterpenoid-related genes sharing high homology with the five candidate *KhCYP450* genes participated in C-22 or C-26 hydroxylation and oxidation. In addition, these five candidates were phylogenetically distant from experimentally verified enzymes in *A. thaliana* responsible for C-22 and C-26 hydroxylation and shared limited sequence homology. This discrepancy was likely caused by differences in substrate backbone structures: Xuetongsu belonged to cycloartane tetracyclic triterpenes; the substrates of their homologous triterpenoid genes were oleanane pentacyclic triterpenes or tirucallane tetracyclic triterpenes, whereas substrates of related enzymes in *A. thaliana* were steroids. Variations in spatial conformation altered the substrate-binding pocket specificity of CYP450s and impeded favorable enzyme–substrate interaction. Nevertheless, the exact physiological functions of these candidate *KhCYP450* genes in Xuetong required further functional characterization via heterologous expression in *Nicotiana benthamiana* or *S. cerevisiae*, as well as CRISPR/Cas9-mediated genome editing in subsequent research.

Moreover, this study had several inherent limitations. First, functional predictions for all candidate genes relied solely on phylogenetic analysis, protein structural simulation, and transcriptome correlation data. Only qRT-PCR expression quantification was performed in this study, while no in vitro enzyme activity assays or in planta transgenic functional validation were conducted. Accordingly, the proposed involvement of these *KhCYP450* candidates in xuetongsu biosynthesis remained purely hypothetical until confirmed by enzymatic assays, heterologous expression systems, or reverse genetics approaches. Second, candidate screening was restricted to genes exhibiting high correlation with Xuetongsu accumulation and root-preferential expression patterns. Genes with low transcript abundance and weak correlation coefficients were excluded from the screening pool, which might result in the omission of potential enzymes responsible for C-22 or C-26 hydroxylation. For future research, we intend to verify the biochemical functions of candidate *KhCYP450* genes through heterologous expression in *N. benthamiana* and *S. cerevisiae*, aiming to clarify their exact catalytic properties and underlying molecular mechanisms. Furthermore, key enzyme genes involved in the Xuetongsu biosynthetic pathway can be used as bait genes for co-expression analysis combined with metabolomic datasets. This approach can further optimize the gene screening workflow, facilitate the precise identification of core functional *KhCYP450* genes that regulate Xuetongsu biosynthesis, and lay a solid theoretical foundation for the resource development and large-scale utilization of Xuetong.

## 4. Materials and Methods

### 4.1. Plant Materials

Plant materials representing six distinct tissue types (roots, leaves, fruits, bark, xylem, and flowers) of wild type Xuetong used in this study were collected from Hupingshan, Shimen County, Hunan Province, China; 29°99′ N, 110°86′ E in August 2023, with three biological replicates and six plants per replicate. These plant materials were identified as *K. heteroclita* by Professor Wei Wang from Hunan University of Chinese Medicine, and the specimens (Accession No. 20160218) were stored in the TCM and Ethnomedicine Innovation & Development International Laboratory at the School of Pharmacy, Hunan University of Chinese Medicine. Immediately after collection, tissues were snap-frozen in liquid nitrogen and maintained at −80 °C in cryogenic storage systems.

### 4.2. Total RNA Extraction and Transcriptome Sequencing

Plant materials from different tissue parts of Xuetong (roots, leaves, fruits, bark, xylem, and flowers) were ground to fine powder in liquid nitrogen, and subsequently, total RNA from different parts was extracted according to the operation process of RNAprep Pure polysaccharide and polyphenol plant total RNA extraction kit (TIANGEN, Beijing, China). RNA purity and concentration were determined by NanoDrop™ One spectrophotometer (Thermo Fisher Scientific, Waltham, MA, USA). RNA integrity was evaluated by RNA Integrity Number (RIN) analysis using an Agilent 5400 Bioanalyzer (Agilent Technologies, Santa Clara, CA, USA). Qualified RNA samples were used for library preparation and transcriptome sequencing, which were performed by Wuhan Frasergen Bioinformatics Co., Ltd. (Wuhan, China). Transcriptome assembly and subsequent analyses including alternative splicing detection, differential expression quantification (FPKM values), and functional annotation were performed using the Xuetong genome established by our research group as a reference.

### 4.3. Identification of KhCYP450s

To characterize CYP450 genes in Xuetong, the Hidden Markov Model (HMM) profile corresponding to the conserved CYP domain (PF00067) was retrieved from Pfam 37.4 (https://pfam-legacy.xfam.org/, accessed on 9 March 2024) [57,58]. The proteome sequences were systematically screened using HMMER v3.4 (http://hmmer.org/, accessed on 5 October 2025) with a significance cutoff of E-value ≤ 1 × 10^−5^ [59]. The obtained amino acid sequence was analyzed using the NCBI Conserved Domain Database (CDD v3.21) (https://www.ncbi.nlm.nih.gov/Structure/bwrpsb/bwrpsb.cgi, accessed on 9 March 2024) in automatic mode. Database searches were performed against CDD-59693PSSM with an E-value cutoff of 0.01, and the options “include retired sequences” and “composition-based statistics” were enabled. The maximum number of hits was set to 500 for comprehensive domain annotation [60,61,62,63]. A total of 449 high-confidence *KhCYP450* transcripts (corresponding to 367 *KhCYP450* genes) were identified (Table S1).

Physicochemical characterization of KhCYP450 proteins (amino acid composition, molecular weight, theoretical isoelectric point (pI), aliphatic index, instability index, and grand average of hydropathicity (GRAVY) was performed using ExPASy-ProtParam (https://www.expasy.org/) [64]. Subcellular localization of KhCYP450s was predicted using the high-quality model of DeepLoc 2.1 (https://services.healthtech.dtu.dk/services/DeepLoc-2.1/, accessed on 7 October 2025) [65], while the transmembrane helices of KhCYP450 proteins were predicted using TMHMM 2.0 (https://services.healthtech.dtu.dk/services/TMHMM-2.0/, accessed on 7 October 2025) [66].

### 4.4. Phylogenetic Analysis, Conserved Motifs, and Gene Structures of KhCYP450s

Protein sequences of *A. thaliana* CYP450 (AtCYP450) were retrieved from The *Arabidopsis* Information Resource (TAIR) database (www.arabidopsis.org) [37]. These sequences were combined with KhCYP450s homologs for multiple sequence alignment using MUSCLE v5 (https://www.drive5.com/muscle5/, accessed on 31 July 2024) [67] under default parameters, and the poorly aligned regions were automatically trimmed using the “automated” parameter in trimAL v1.4.1 (https://vicfero.github.io/trimal/, accessed on 1 August 2024) [68]. The trimmed alignment was subsequently used to construct a maximum likelihood (ML) tree using IQ-TREE v2.3.6 (http://www.iqtree.org/) [69,70,71]. The best-fit evolutionary model (Q.plant + R8) was determined by the integrated ModelFinder tool, and tree robustness was evaluated through ultrafast bootstrap analysis with 1000 replicates. Finally, the phylogenetic tree was visualized and functionally annotated using EvolView v4 (https://www.evolgenius.info/, accessed on 1 August 2024) [72].

Conserved protein motifs were identified through MEME Suite v5.5 [73,74] (https://meme-suite.org/, accessed on 17 August 2024) under stringent parameters: minimum motif width = 6; maximum motif width = 50; number of motifs to find = 10. The gene structure of *KhCYP450s*, including coding sequences (CDS), untranslated regions (UTR), introns, and exons, was extracted from the Xuetong genome annotation in GFF3 format. The conserved motifs and gene structure were visualized and analyzed using TBtools v2.084 [75].

### 4.5. Synteny Analysis, Gene Duplication and Chromosomal Localization of KhCYP450s

Gene duplication events and syntenic relationships among *KhCYP450s* were analyzed using the One Step MCScanX tool in TBtools-II v2.084 [75] with a stringent E-value threshold (1 × 10^−10^). Tandem duplication pairs and Whole Genome Duplication (WGD) or Segmental duplication pairs were visualized through the Advanced Circos module, which simultaneously mapped genome-wide gene density distribution using quantile-based interval partitioning. The Simple Ka/Ks Calculator (NG model) plugin in TBtools was used to compute the nonsynonymous (Ka) and synonymous (Ks) substitution rates of duplicated gene pairs and to estimate the selective pressure based on the Ka/Ks ratio. Chromosomal localization of *KhCYP450s* within the Xuetong genome was also conducted using TBtools.

### 4.6. Cis-Acting Element Analysis in Promoters

The 2000 bp region upstream of the 5′ UTR from each *KhCYP450* transcript was designated as promoter sequences. These promoter sequences were extracted with TBtools-II v2.084, and cis-acting elements were predicted using PlantCARE (https://bioinformatics.psb.ugent.be/webtools/plantcare/html/, accessed on 10 October 2024) [76]. Visualization of the predicted cis-acting elements was performed using GraphPad Prism v10.1.2.

### 4.7. Gene Function Annotation of KhCYP450s

Functional annotation was performed via the online eggNOG platform (http://eggnog-mapper.embl.de/, accessed on 15 October 2024) [77,78], and visualized using Graphpad prism v10.1.2.

### 4.8. Weighted Gene Co-Expression Network Analysis (WGCNA) of KhCYP450s

WGCNA was a systems biology method employed to explore the relationships among multiple genes and calculate their associated traits [79,80]. The co-expression network of *KhCYP450s* genes in Xuetong was constructed using WGCNA version 1.73 [81]. Initially, the FPKM values (Table S17) of all *KhCYP450* genes derived from the transcriptomic data were imported into R software for preprocessing, and genes with expression levels greater than 0 in at least 10% of samples were retained for further analysis, leaving a total of 356 *KhCYP450s* for subsequent WGCNA. After detecting missing values, sample clustering was performed based on average expression, and potential outliers were identified and visualized in a sample dendrogram. An unsigned topological overlap matrix (TOM) was used to construct the co-expression network and module identification with the following parameters: ablinehigh = 0.8, softPower = 11, minimum module size = 30, and mergeCutHeight = 0.25, ensuring robust grouping of all genes into distinct modules. The association between each sample trait and the identified modules was subsequently assessed using Pearson correlation analysis.

### 4.9. Extraction and Quantification of Xuetongsu

A total of 1.000 g of dried sample was accurately weighed and transferred into a 50 mL stoppered Erlenmeyer flask, and 20 mL of 80% acetonitrile was added for extraction. Ultrasonic treatment was conducted at room temperature for 60 min. After cooling, the solvent volume was supplemented to compensate for evaporation loss. The extract was filtered, and the resulting supernatant was passed through a 0.22 μm microporous membrane. A 10 μL aliquot of the filtrate was loaded into an HPLC vial, and all samples were analyzed in triplicate.

HPLC analysis was performed using an Eclipse XDB C-18 analytical column (4.6 × 250 mm, 5 μm) fitted with a matching Eclipse XDB C-18 guard column (20 × 4 mm, 5 μm) (Agilent Technologies, Santa Clara, CA, USA). The column temperature was maintained at 30 °C, and the detection wavelength was set at 210 nm. The mobile phase comprised acetonitrile (A) and 0.05% aqueous phosphoric acid (B). The gradient elution procedure was as follows: 60–95% A from 0 to 30 min, followed by isocratic elution with 95% A for 30–35 min. The column was re-equilibrated for 10 min after each run. The flow rate was 1.0 mL/min with an injection volume of 10 μL. The standard curve for the xuetongsu reference standard was determined as *y* = 11.838*x* + 6.8435, *R*^2^ > 0.9999.

### 4.10. Gene Expression Analysis by qRT-PCR

CYP450 mRNA expression levels across Xuetong tissues were analyzed by quantitative real-time PCR (qRT-PCR) using a QuantStudio™ 7 Flex Real-Time PCR System (Applied Biosystems, Foster City, CA, USA). Total RNA was reverse-transcribed into cDNA using ReverTra Ace™ qPCR RT Master Mix with gDNA Remover (Toyobo BioScience, Shanghai, China). qRT-PCR was performed using Hieff^®^ qPCR SYBR Green Master Mix (Low Rox Plus) (Yeasen Biotechnology, Shanghai, China). According to our previous studies, *UBC* was selected as the reference gene [51]. All samples were analyzed with three technical replicates and three biological replicates. The relative expression levels of the genes were quantified based on the Ct values obtained from qRT-PCR analysis via the 2^−ΔΔCt^ method [82]. Data visualization employed GraphPad Prism v10.1.2. All primers designed using Primer Premier 6.0 are listed in Table S14 and were synthesized by Sangon Biotech (Shanghai) Co., Ltd. (Shanghai, China).

### 4.11. Statistical Analysis

Statistical analyses were performed in R version 4.2.1. Data normality for Xuetongsu content across tissues was verified by Shapiro–Wilk testing. Spearman’s rank correlation coefficients with Benjamini–Hochberg adjusted *p*-values were calculated to assess the association between Xuetongsu concentrations and *KhCYP450s* expression profiles. Pearson correlation analysis was subsequently conducted to evaluate expression level concordance between RNA-seq and qRT-PCR datasets, with statistical significance determined at α = 0.05.

### 4.12. Analysis of Protein Structure

The UniProtKB Swiss-Prot database was selected through the UniProt platform (https://www.uniprot.org/, accessed on 1 August 2025) [83]. BLAST alignment was performed using the built-in UniProt BLAST with default parameters, and precomputed AlphaFold structural models corresponding to the BLAST alignment hits were subsequently retrieved from the UniProt interface. The three-dimensional (3D) structure of KhCYP450s proteins was predicted using the AlphaFold3 tool (https://alphafoldserver.com/, accessed on 2 August 2025) [84]. Structural quality assessment was conducted via the SWISS-MODEL server (https://swissmodel.expasy.org/assess, accessed on 2 August 2025) [85], employing evaluation parameters including Ramachandran plot, membrane detection, stereochemistry, and model quality estimation. The CASTpFold platform (https://cfold.bme.uic.edu/castpfold/, accessed on 3 August 2025) [86] was implemented for active site prediction with a probe radius parameter of 1.4 Å. PyMOL v3.0.3 software [87] was utilized for 3D structural analysis and visualization, where structural superposition was achieved through the “align” command. Quantitative comparisons included calculation of the Euclidean distance between geometric centers of paired active sites and determination of atomic overlap proportions within a 3 Å threshold. Bidirectional overlap ratios were computed independently and subsequently averaged.

## 5. Conclusions

In this study, 367 CYP450 genes were identified in Xuetong, which were categorized into nine distinct clans. *KhCYP450s* clustered in the CYP71, CYP72, and CYP85 clans were more likely to participate in triterpenoid modification. Several key genes, including *Khe09540.1*, *Khe21316.1* and *Khe22992.1*, shared high sequence homology with functionally characterized triterpenoid biosynthetic enzymes and harbored conserved structural features, suggesting their potential catalytic roles in the oxidative modification of the triterpenoid skeleton during xuetongsu biosynthesis. Overall, this investigation advanced the understanding of the CYP450 superfamily in Xuetong and established a critical foundation for elucidating the mechanisms of Xuetongsu biosynthesis.

## Data Availability

The datasets generated and analyzed during the current study are available in the Zenodo repository, [https://doi.org/10.5281/zenodo.17284212]. The transcriptome raw data of different tissues are available in the China National Center for Bioinformation (https://ngdc.cncb.ac.cn/gsa/, accessed on 27 May 2025) repository [CRA026237].

## Supplementary Materials

The following supporting information can be downloaded at: https://www.mdpi.com/article/10.3390/molecules31122140/s1, Figure S1: Schematic diagram of conserved domains in KhCYP450s. The protein sequence of each CYP450 was verified using the NCBI Conserved Domain Database tool; Figure S2: Schematic diagram of conserved motifs in KhCYP450s. Different motifs were represented by different colors; Figure S3: WebLogos of the 10 conserved motifs in KhCYP450s. Each letter represented an amino acid, and its height corresponded to the relative frequency; Figure S4: Schematic diagram of gene structures in *KhCYP450s*. Green and orange rectangles represented untranslated regions (UTR) and coding sequences (CDS), respectively. The lines without any rectangles represented introns; Figure S5: Chromosomal distribution of *KhCYP450s*. The left scale indicated the length (Mb) of Xuetong chromosomes; Figure S6: Number of cis-acting elements in the promoter region of nine clans of *KhCYP450s*; Figure S7: Schematic diagram of categories of cis-acting elements in the promoters of *KhCYP450s*; Figure S8: Content of Xuetongsu in different tissues in Xuetong ($\bar{x}$ ± *s*, *n* = 10). Lowercase letters indicated significant differences among different tissues at the *p* < 0.05 level, while uppercase letters indicated significant differences among different tissues at the *p* < 0.01 level; Table S1: Correspondence between transcript IDs and gene IDs of the *KhCYP450* superfamily; Table S2: Detailed information of the 367 *KhCYP450s*. The contents included gene accession numbers, chromosomal locations, gene ranges and lengths, family and clan classifications based on CYP nomenclature and phylogenetic tree, numbers of exons, introns, CDS and UTRs, duplication types, as well as physicochemical properties such as amino acid number, molecular weight, theoretical isoelectric point, subcellular localization, instability index, aliphatic index and grand average of hydropathicity; Table S3: CYP450s proteins used for phylogenetic analysis; Table S4: Detailed information on seven triterpenoid- and steroid-related CYP450 genes from *Arabidopsis thaliana*; Table S5: Classification of Tandem duplicated genes; Table S6: Tandem and WGD or Segmental duplicated gene pairs in *KhCYP450s*. Bold fonts indicated genes that underwent both duplication events. The Ka/Ks ratio of each duplicated pair was calculated; Table S7: Classification of cis-acting elements in the promoters of *KhCYP450s*; Table S8: Analysis of cis-acting elements in the promoters of *KhCYP450s*; Table S9: GO term analysis of *KhCYP450s*; Table S10: KEGG pathway analysis of *KhCYP450s*; Table S11: Module assignments and membership (kME) values of *KhCYP450s* in WGCNA; Table S12: The Correlation between *KhCYP450s* RNA-seq and Xuetongsu content; Table S13: Summary of candidate *KhCYP450s* potentially involved in Xuetongsu biosynthesis. A total of 35 *KhCYP450s* showed significant correlations (*R* > 0.75, *p* < 0.01) between gene expression levels and Xuetongsu content. Information on family classification, WGCNA modules, tissue-specific expression profiles and correlation coefficients was provided; Table S14: Primers used for qRT-PCR; Table S15: Structural alignment and active site analysis of KhCYP450s with functionally characterized proteins; Table S16: Quality assessment of three-dimensional structures of KhCYP450s proteins; Table S17: FPKM values of *KhCYP450s* across different tissues.
