# Supplementary figures and images for: Insights into the Cytochrome P450 Monooxygenase Superfamily in Kadsura heteroclita (Xuetong)
Source: Molecules. 2026 Jun 17;31(12):2140. doi: 10.3390/molecules31122140 (PMC13305425; doi:10.3390/molecules31122140)

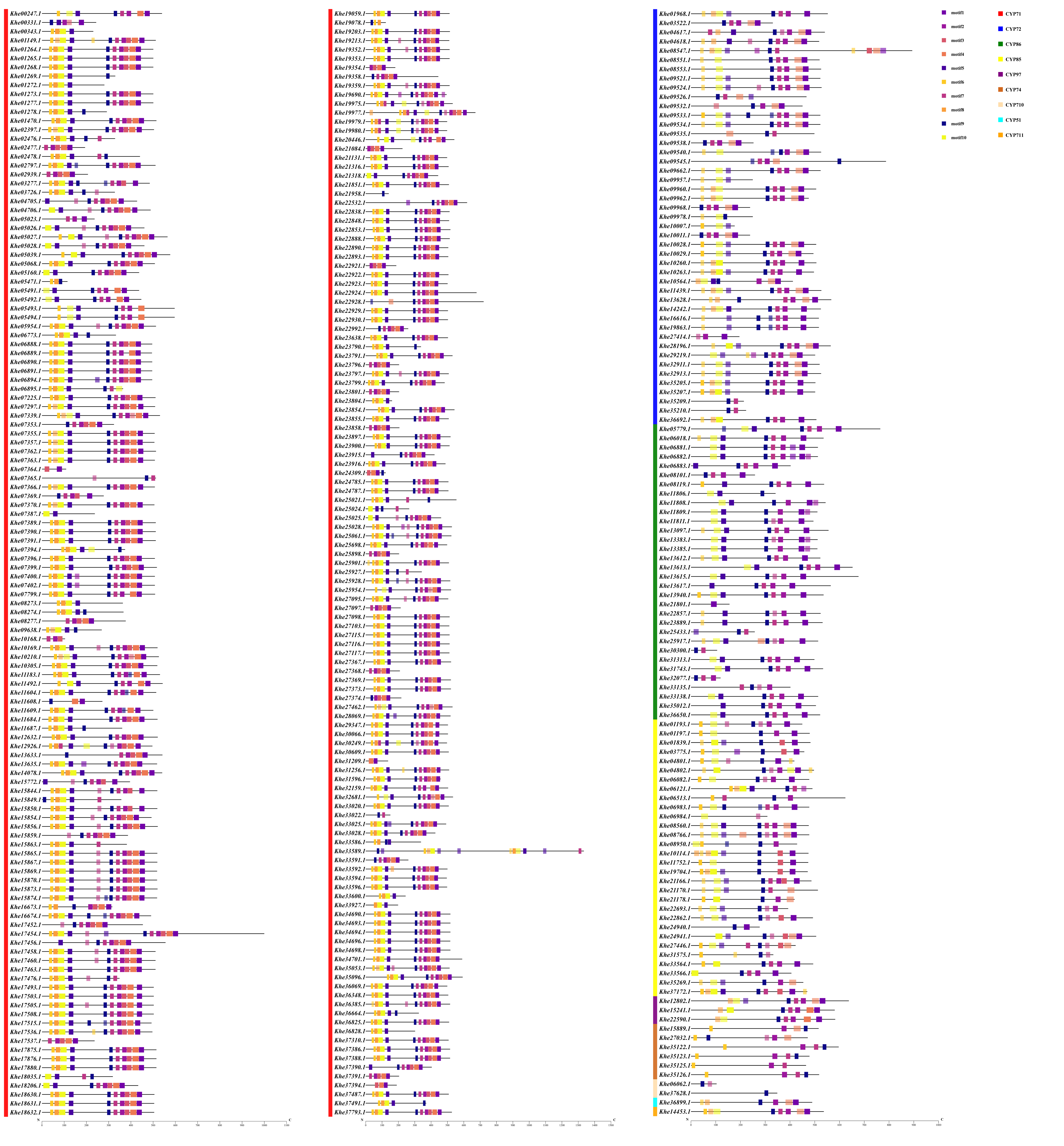

Supplement: Supplementary file 1 [file molecules-31-02140-s001.zip › Supplementary Files/Supplementary Figure S2.tif]

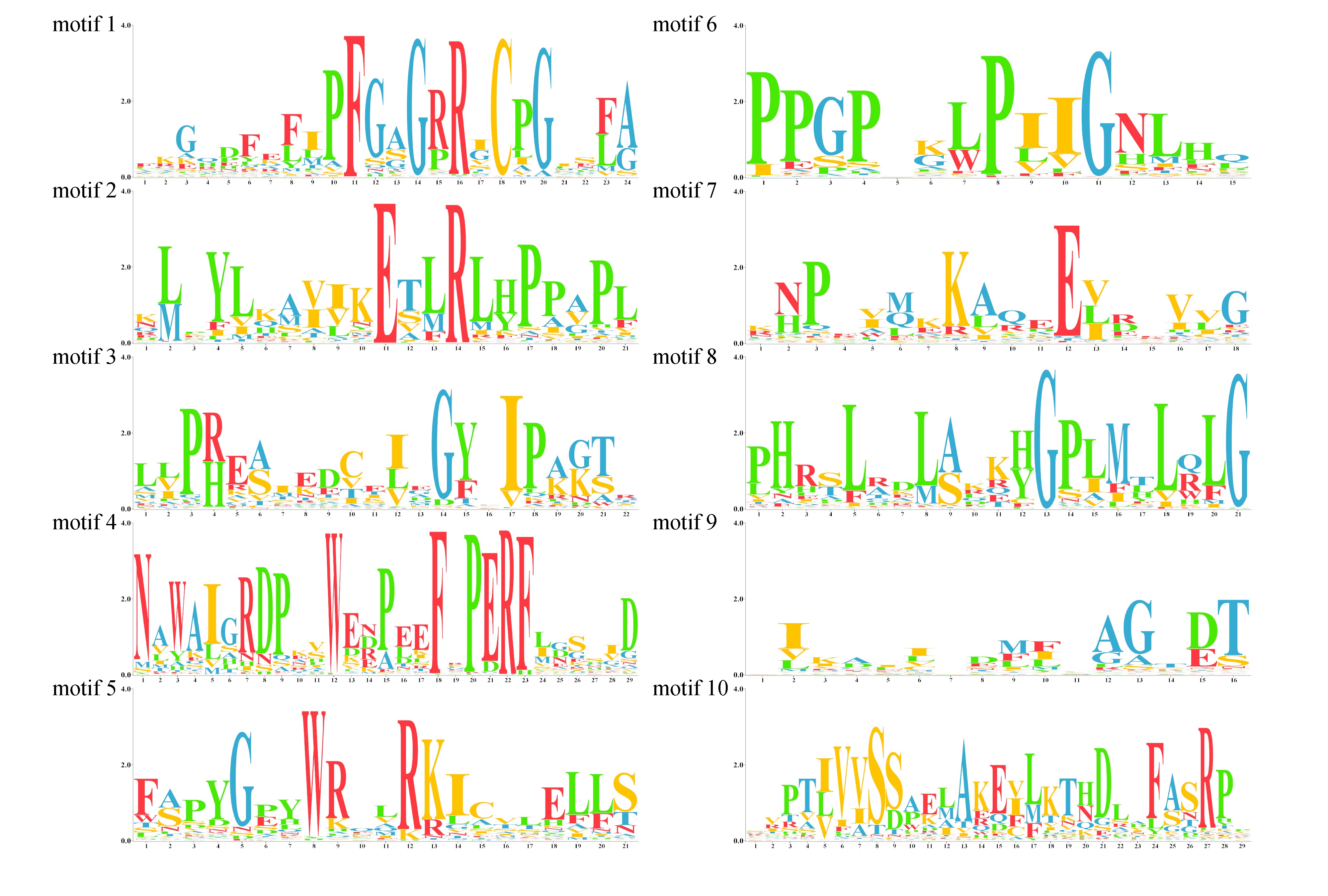

Supplement: Supplementary file 1 [file molecules-31-02140-s001.zip › Supplementary Files/Supplementary Figure S3.jpg]

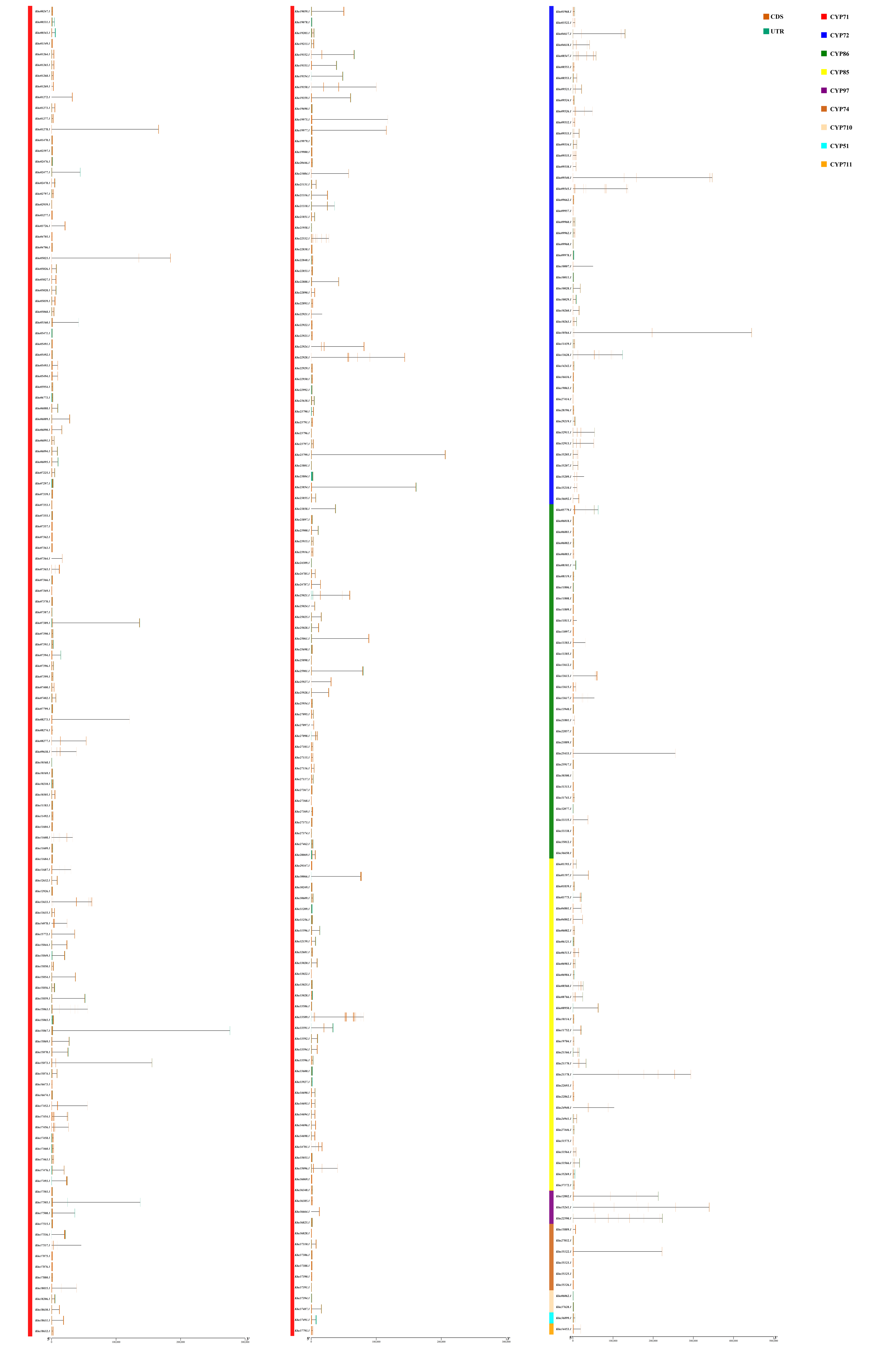

Supplement: Supplementary file 1 [file molecules-31-02140-s001.zip › Supplementary Files/Supplementary Figure S4.tif]

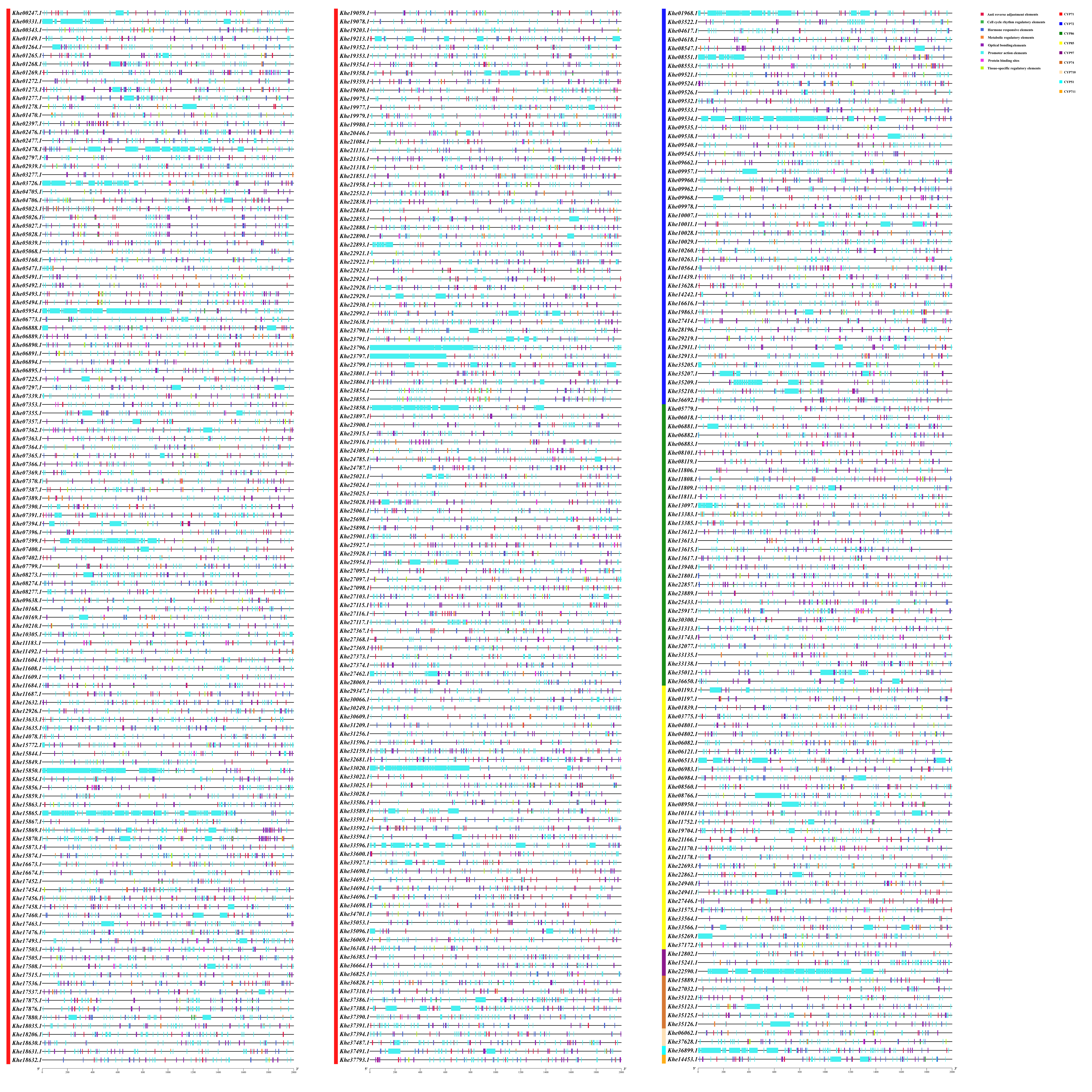

Supplement: Supplementary file 1 [file molecules-31-02140-s001.zip › Supplementary Files/Supplementary Figure S7.tif]
